# Supplementary material for: Sequence-regulated copolymerization based on periodic covalent positioning of monomers along one-dimensional nanochannels
Source: Nat Commun. 2018 Jan 23;9:329. doi: 10.1038/s41467-017-02736-1 (PMC5780473; doi:10.1038/s41467-017-02736-1)
Supplement: Supplementary file 1 — Supplementary Information [file 41467_2017_2736_MOESM1_ESM.pdf]

Supplementary Methods

Materials. All of the reagents and chemicals used were obtained from commercial sources, unless otherwise noted. 2,2′-Azobis(isobutyronitrile) (AIBN) was recrystallized from MeOH solution. Acrylonitrile (**A**) and methyl vinyl ketone (**M**) were purified by vacuum distillation prior to use. Styrene-3,5-dicarboxylic acid (**S**) was synthesized according to previously described method.[1]

Measurement. Single-crystal X-ray diffraction data for **1S**⊃H_2O were collected at 95(2) K using synchrotron radiation on the BL40XU instrument at SPring-8 utilizing a high-precision diffractometer with a Rigaku Saturn 724 CCD camera. The synchrotron radiation was monochromated using a Si(111) channel-cut monochromator (λ = 0.78224 Å). The structure was solved by a direct method (SHELXS-97) and refined by full-matrix least-squares procedures on F^2 for all reflections (SHELXL-2014). The hydrogen atoms were positioned geometrically and refined using a riding model. The electron densities of the disordered guest molecules were flattened using the SQUEEZE option of PLATON.[2,3] The deposited number of Cambridge Crystallographic Data Centre (CCDC) is 1430041 for **1S**⊃H_2O. X-ray powder diffraction (XRPD) data were recorded on a Rigaku SmartLab X-ray diffractometer with Cu Kα radiation. Gel permeation chromatography was performed in DMF at 40 °C on three linear-type polystyrene gel columns (Shodex K-805L) that were connected to a Jasco PU-980 precision pump, a Jasco RI-930 refractive index detector and a Jasco UV-970 UV−vis detector set at 256 nm. The ^{1}H NMR spectra were obtained using JEOL A-500 and ECS-400 spectrometers operating at 500 and 400 MHz, respectively. The ^{13}C NMR spectra were obtained using a JEOL ECA-600P spectrometer operating at 600 MHz. The IR spectra were measured employing a Thermo Scientific Nicolet iS5. Nitrogen adsorption at 77 K was measured using BELSORP-mini equipment. Before the adsorption measurement, the sample was treated under high vacuum ($<10^{-2}$ Pa) at 110 °C for 2 h. Elemental analyses were recorded on a J-Science JM 10. Experiments using microwaves were conducted in a Biotage Initiator$^+$. SEM measurements were performed using a Hitachi S-3000N at an accelerating voltage of 5 kV. Samples were put on a conducting carbon tape attached by an SEM grid, and then coated with platinum. Particle size distributions were obtained using a Horiba Partica LA-950 laser diffraction particle size analyser.

Recrystallization of 1S after copolymerization process. After the isolation process of **P1** (35 mg) from **1S** (168 mg), the ethanol/water solution of residual **1S** was collected and then evaporated. Deficient amount of **S** (19.1 mg, 0.099 mmol) was added to the mixture in order to equalize the molar amount of **S** and Cu, followed by the addition of methanol (13 mL). The equivalent molar of pyridine (0.0529 μL, 0.662 mmol) against Cu ion was then dropped into the solution, and the mixture was stirred for 12 h. The mother liquor was decanted and resulting light blue solid was washed with methanol to remove unreacted materials. Drying the solid under vacuum for 2 h at room temperature gave **1S**⊃H_2O (27 mg).

Copolymerization of A with S in DMF. A typical procedure is as follows. Calculated amounts of **A** and **S** monomers were mixed with AIBN (1 mg) in dry DMF (1 mL). The reaction mixture was irradiated with UV light under a nitrogen atmosphere to initiate the polymerization. Finally, the reaction mixture was poured into MeOH to give copolymer as a precipitate.

Methylation of P1. P1 (13 mg) was added to a solution of *N*,*N*-dimethylformamide dimethylacetal (0.096 mL) in dry DMF (0.39 mL) in a reaction tube and sealed under nitrogen atmosphere. The reaction tube was irradiated in a microwave reactor with a slow ramp at 140 °C over 10 min and then maintained for an additional 50 min. After cooling to room temperature, H_2O was added to the reaction mixture and it was centrifuged for washing three times. The sample was collected and dried under reduced pressure (Supplementary Fig. 12).

Simulation model for 1S⊃A with propagating radical. Molecular dynamics (MD) simulations were performed using the general AMBER force field (GAFF). Atom types in GAFF were determined by an antechamber program in the AMBER 12 package. The atomic charges were determined by the Merz–Kollman scheme at the B3LYP/6-31+G(d,p) level of density functional theory (DFT) calculations. For DFT calculations including **S** monomer, each of the carboxylic groups was capped with a Li^+ to mimic the coordination of **S** to the copper cations in the PCP framework, and the atomic charges of the Li^+ were distributed to the oxygen atoms to keep the molecules neutral. By fitting the optimized structures and rotational barriers of **SA**$^\cdot$ and **AS**$^\cdot$ models at the B3LYP/6-31G(d) level of DFT calculations, several parameters were modified or created. DFT calculations of radical molecules were performed in the doublet state with unrestricted open shell treatment.

Replica-exchange molecular dynamics (REMD) simulations of propagating radicals and free guest **A** monomers in the hexagonal channel of **1S** were performed. During the simulations, oxygen atoms and the carbon and hydrogen atoms at the 2-position of **S** were kept fixed to maintain the channel structure by harmonic-oscillator potentials. This approximation can be justified by the fact that the fixed atoms are not accessible to both the propagating radicals and **A** monomers because of the steric hindrance from adjacent styryl groups. The initial structural model for **1S⊃A** during copolymerization was prepared with a periodic boundary condition. This model was composed of the hexagonal channel (34 **S** monomers) and guest species (18 **A** monomers and a Me**AASAAASAA**$^{\bullet}$ chain). In this simulation, Me**AASAA**$^{\bullet}$ was placed in the hexagonal channel, followed by an *NVT* (constant volume and temperature) MD simulation for 10 ps at 343 K. Then, we created a new bond between the carbon radical and the tail carbon atom of monomer with the shortest head–tail distance ($r^{\mathrm{R–M}}$). This procedure was repeated four times to make the Me**AASAAASAA**$^{\bullet}$ model chain (Supplementary Fig. 16). The periodic boundary box size was $x \times y \times z = 24.0 \times 24.0 \times 41.0$ Å^3, and the channel along the *z*-axis (in the *c*-axis direction of the crystal structure) was connected with the adjacent channels.

Simulation for reactivity. Nanosecond time-scale MD simulations were first performed. However, no dihedral angle rotation of the terminal group in the propagating radicals took place during the simulations due to the conformational restriction in the PCP nanochannel. Thus, we adopted the REMD method, which efficiently samples large regions of the phase space and can also analyse many conformations of the radical terminals. The number of replicas was 36 with temperatures ranging from 331 to 1200 K, and the trajectories of the replica at 343 K were analysed. Replica temperature exchanges were attempted every 1 ps and repeated 15000 cycles for each analysis. Here, we arbitrarily assumed that the monomer can react with the radical when $r^{\mathrm{R–M}}$ is less than 3.2 Å because of the shorter distance than the sum of the van der Waals (vdW) radius parameters of the carbon atoms (3.816 Å).

Reaction barrier for the reaction of ~SA$_{n}$ radicals with monomers. Reaction barriers of the elongation step were calculated by optimizing the reactant (fixing $r^{\mathrm{R–M}}$ = 3.2 Å), transition state (TS) and product structures. The level of DFT calculations was M06-2X/6-311+G(2df,p)//B3LYP/6-31G(d). As the DFT calculation model, snapshots from the REMD trajectory with the shortest $r^{\mathrm{R–M}}$ were chosen, and the ~**SA**$_{n}^{\bullet}$ terminal contacting with **A** or **S** at the same face of the channel were extracted. To simplify the

model, each of the carboxylic groups was substituted by a hydrogen atom. Test calculations revealed that this simplification resulted in less than 0.2 kcal/mol difference in relative energies between the reactant, TS and product structures. The substituted CH atoms were fixed during the optimizations.

Supplementary Table 1. Single-crystal X-ray data of **1S**⊃H_2O.

**Formula**	$C_{10}H_8CuO_5$
**Formula weight**	271.71
**Temperature (K)**	95(2)
**Wavelength (Å)**	0.78224
**Crystal system**	trigonal
**Space group**	$P\bar{3}m1$
a **(Å)**	18.719(5)
c **(Å)**	6.8137(19)
V **($Å^3$)**	2067.6(13)
Z	6
**Calcd Density (g/cm^3)**	1.309
**μ (mm^{-1})**	2.056
***F*(000)**	822
**Crystal size (mm^3)**	0.04 × 0.02 × 0.01
**Total reflection**	10855
**Unique reflection**	1341
**R_{int}**	0.0992
**Goodness-of-fit**	1.170
**Final R_1 and wR_2 indices [$I > 2\sigma(I)$]^b**	0.0587, 0.1519
**R_1 and wR_2 indices (all data)^c**	0.0810, 0.1773
**max, min $\Delta\rho$ ($eÅ^{-3}$)**	0.776, -1.128
**CCDC number**	1430041

[a] Data based on the PLATON/SQUEEZE[2,3] model. [b] $R_1 = R = \Sigma ||F_o| - |F_c|| / \Sigma |F_o|$. [c] $wR_2 = [\Sigma w(F_o^2 - F_c^2)^2 / \Sigma w(F_o^2)^2]^{1/2}$.

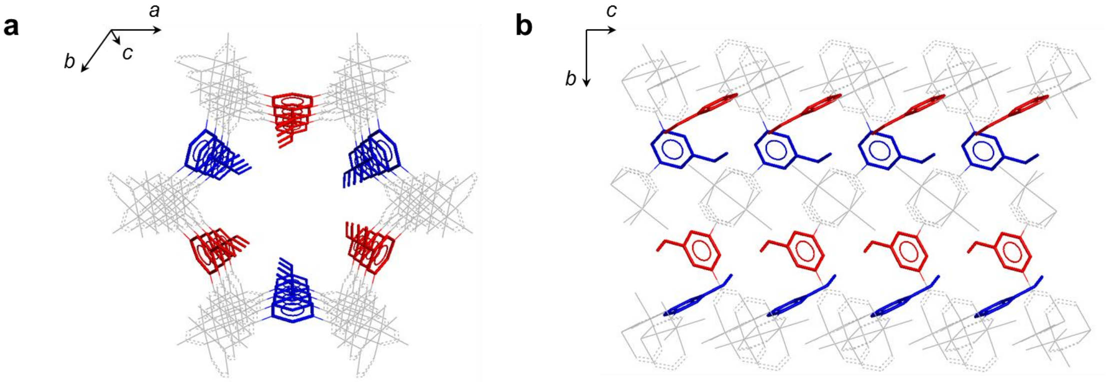

Supplementary Figure 1. View of the hexagonal channel of **1S**⊃H_2O along the (a) *c*- and (b) *a*-axis. Styryl groups are immobilized along the *c*-axis on each face of the channel, while styryl groups at adjacent faces (blue and red) of the channel point in opposite directions. Copper and oxygen atoms are coloured grey, and hydrogen atoms and one of the disordered vinyl moieties are omitted for clarity.

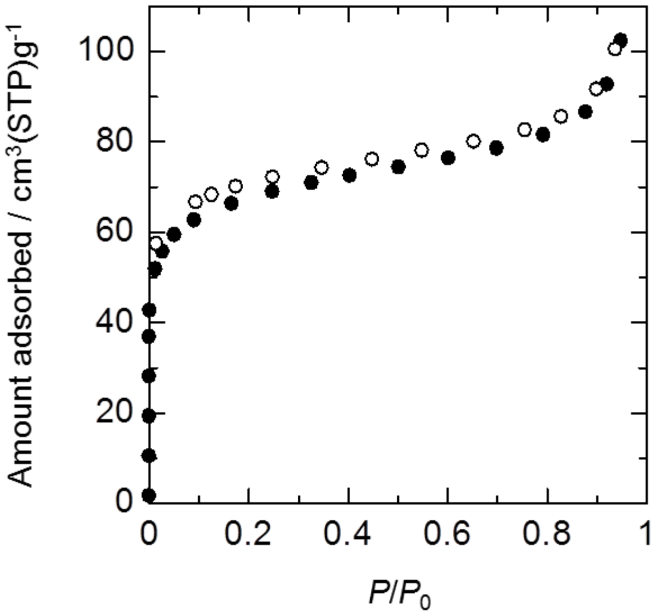

Supplementary Figure 2. Nitrogen adsorption and desorption isotherms of **1S** at 77 K. Filled and open circles represent adsorption and desorption, respectively. The adsorption behaviour was similar to that observed on Cu(5-azidoisophthalate), which has the same topology as **1S**.[4]

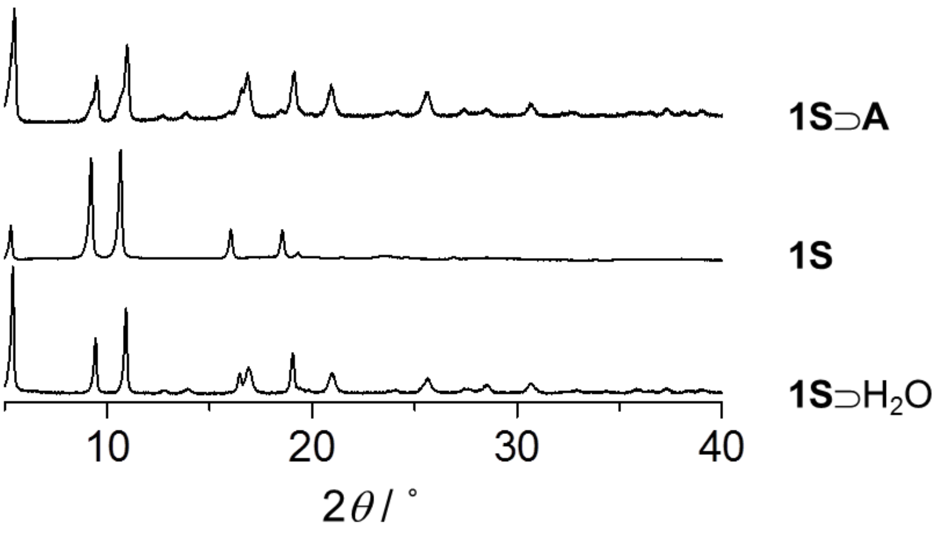

Supplementary Figure 3. XRPD patterns for **1S⊃A**, **1S** and **1S⊃**H_2O. Treatment of **1S⊃**H_2O at 110 °C for 2 h induced the removal of water, giving **1S** as a dehydrated host.[4] After the incorporation of **A** into **1S**, the host structure returned to the original hydrated structure, as shown by the peak positions in the diffractogram of **1S⊃A** being the same as in **1S⊃**H_2O.

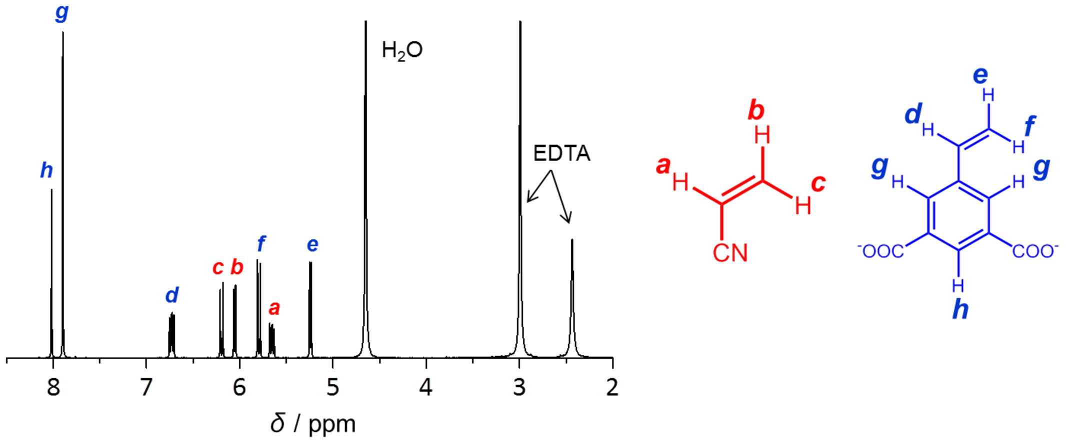

Supplementary Figure 4. ^{1}H NMR spectrum of **1S⊃A** dissolved in D_2O/Na_4-EDTA. **A**/**S** ratio in **1S⊃A** was calculated to be 42/58 from the integral ratio of the resonances corresponding to protons *a* and *d*.

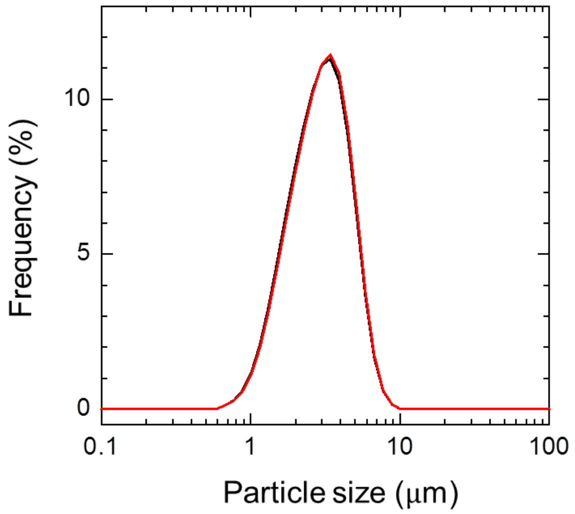

Supplementary Figure 5. Particle size distributions of PCP crystals. The black and red lines represent **1S** and **1S⊃A** after the polymerization, respectively. The size distribution of the original particles was almost the same to that of the sample after the polymerization, suggesting that polymerization reaction did not proceed outside the host crystals.

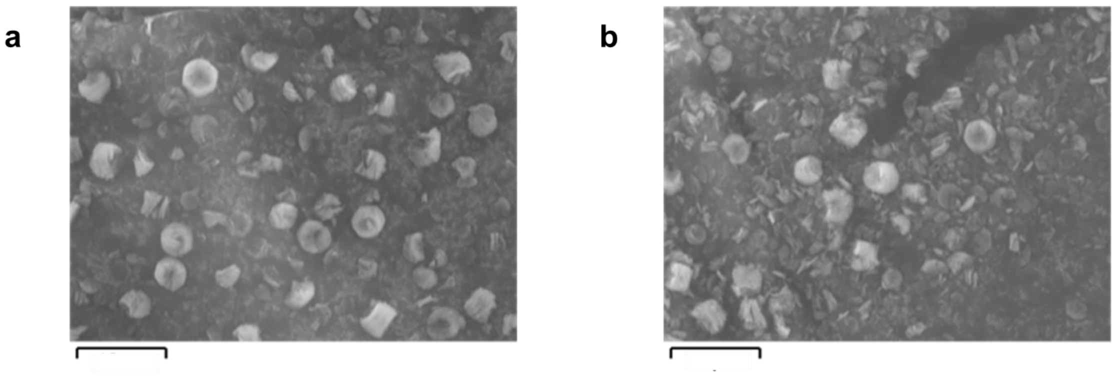

Supplementary Figure 6. SEM images of PCP crystals. (a) and (b) shows the images of **1S** and **1S⊃A** after the polymerization, respectively (scale bar = 10 µm). The size and morphology of the host crystals did not change during the polymerization process. Aggregation of the particles and the deposition of polymeric compound on the crystal surfaces were not observed in the SEM images, indicating that the copolymerization proceeded only inside the pores.

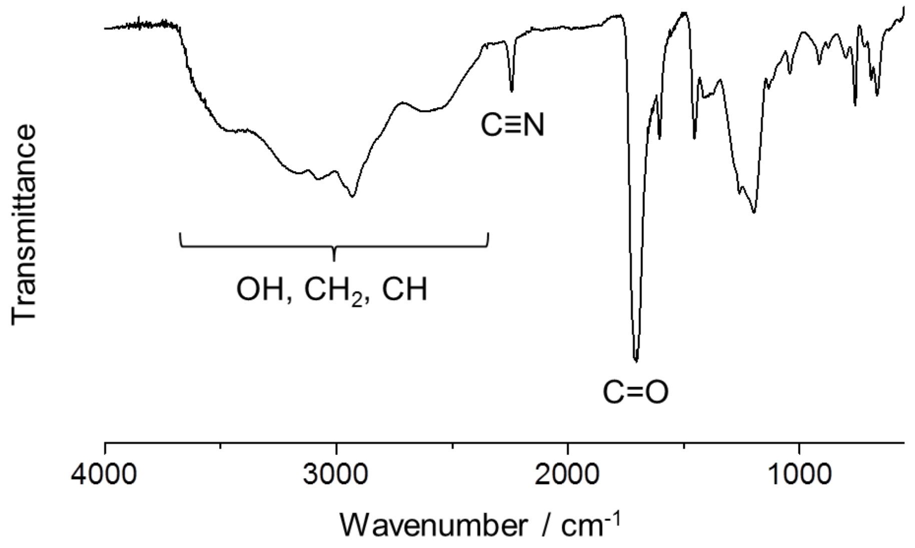

Supplementary Figure 7. IR spectrum of **P1**. Characteristic peaks for C=O, C≡N and O–H stretching vibrations were observed at 1713, 2244, and 2345–3700 cm^{-1}, respectively, indicating that **P1** contained both **A** and **S** units in its structure.

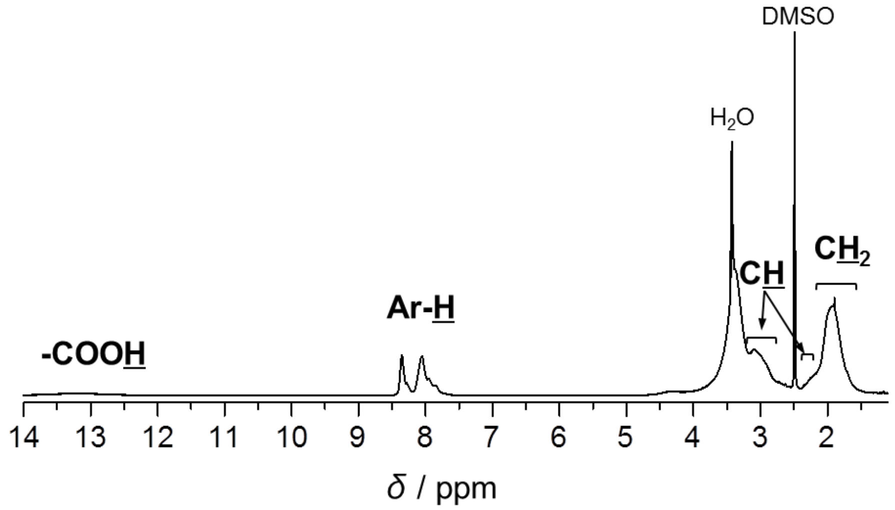

Supplementary Figure 8. ^{1}H NMR spectrum of **P1** in DMSO-d_6. The methylene and methine groups were assigned by DEPT 135 and HMQC spectra (Supplementary Figs. 9 and 10). The copolymer composition of **P1** was determined by comparing the relative integrations of the aromatic protons and methylene protons (**A**/**S** = 75/25), which was consistent with the C/N atomic ratio obtained in elemental analysis of **P1**.

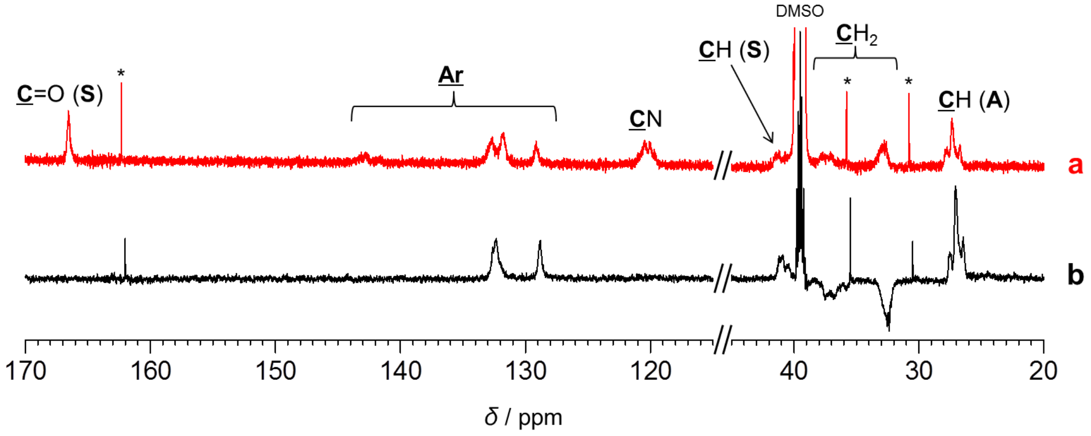

Supplementary Figure 9. (a) ^{13}C NMR and (b) DEPT 135 spectra of **P1**. On the DEPT-135 measurement, primary and secondary carbons show positive and negative phasing peaks, respectively, and neither quaternary carbons nor the carbonyl peaks appear. The cyano, aromatic and carbonyl carbons were assigned based on the chemical shifts. The location of the CH carbon of the **A** unit was consistent with that of a homopolymer of **A**. Asterisks designate DMF.

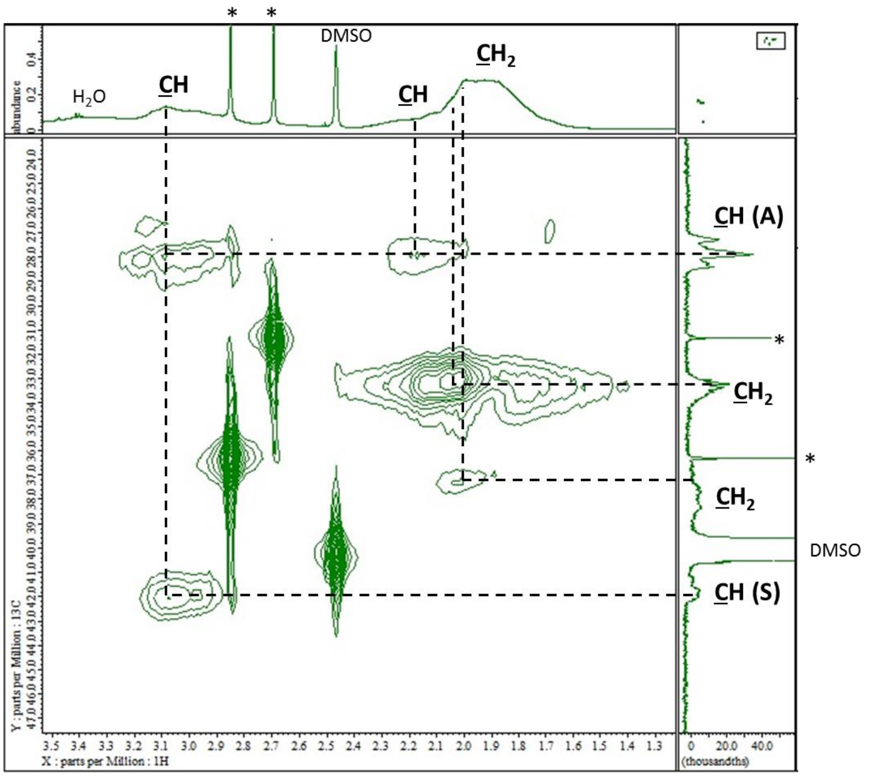

Supplementary Figure 10. HMQC spectrum of **P1**. Cross peaks for methylene and methine protons were observed. Asterisks designate DMF.

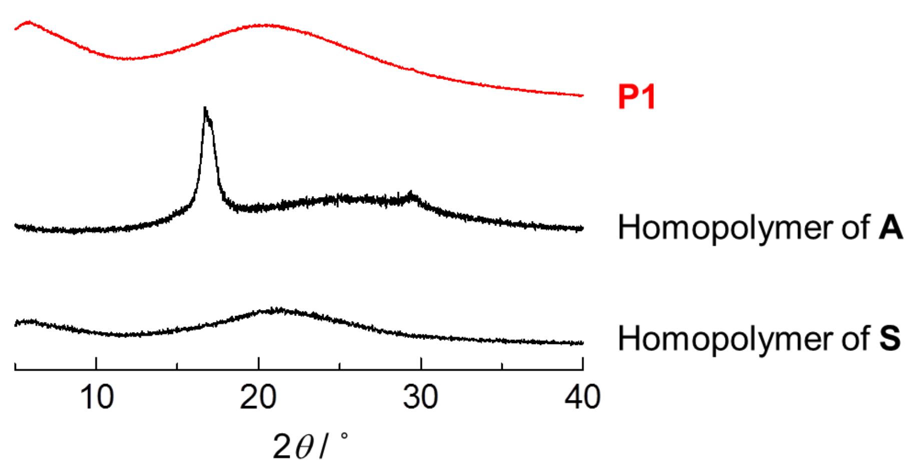

Supplementary Figure 11. XRPD patterns of **P1** and the homopolymers of **A** and **S**. **P1** exhibited no characteristic peak corresponding to the homopolymer of **A**, showing that homopolymerization of acrylonitrile did not proceed in **1S⊃A**.

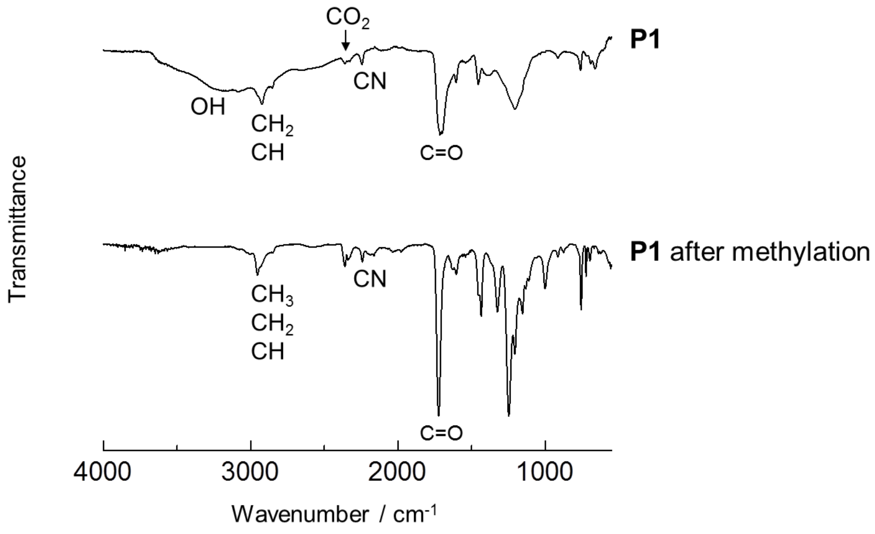

Supplementary Figure 12. IR spectra of **P1** before and after the methylation reaction. A peak for O–H stretching was not observed after the reaction, indicating the full methylation of the carboxy groups of **P1**.

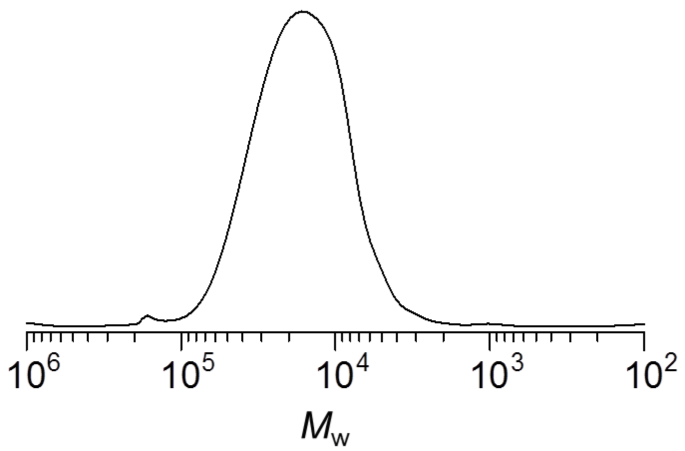

Supplementary Figure 13. GPC profile of methylated **P1** (M_n = 20,000, M_w/M_n = 1.6). Monomodal GPC profile indicated that **P1** was not a mixture of poly(**A**-*co*-**S**) and homopolymers (poly**A** and poly**S**).

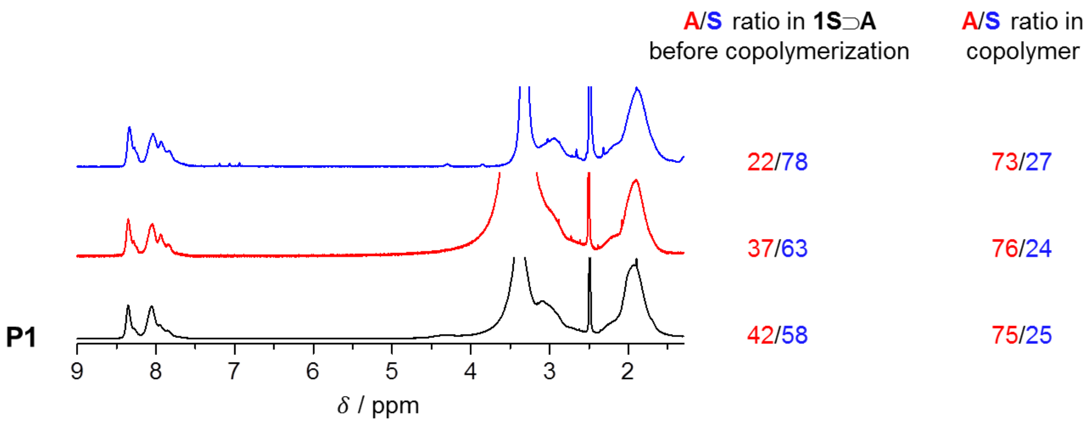

Supplementary Figure 14. ^{1}H NMR spectra of copolymers obtained from **1S**. Copolymers were prepared from **1S⊃A** at different initial monomer feed ratios. The monomer compositions in the polymers were determined by comparing the relative integrations of the aromatic protons and methylene protons (Supplementary Fig. 8). Aromatic proton peaks of **S** in these copolymers were only detectable at a lower magnetic field (δ = 7.8–8.4 ppm), suggesting the distributions of solitary **S** units in continuous **A** linkages (Fig. 4). Despite the different initial monomer ratios, **A**/**S** ratios in the copolymers are almost constant (close to 3/1), indicating the predominant **SAAA** sequence in the copolymer chains.

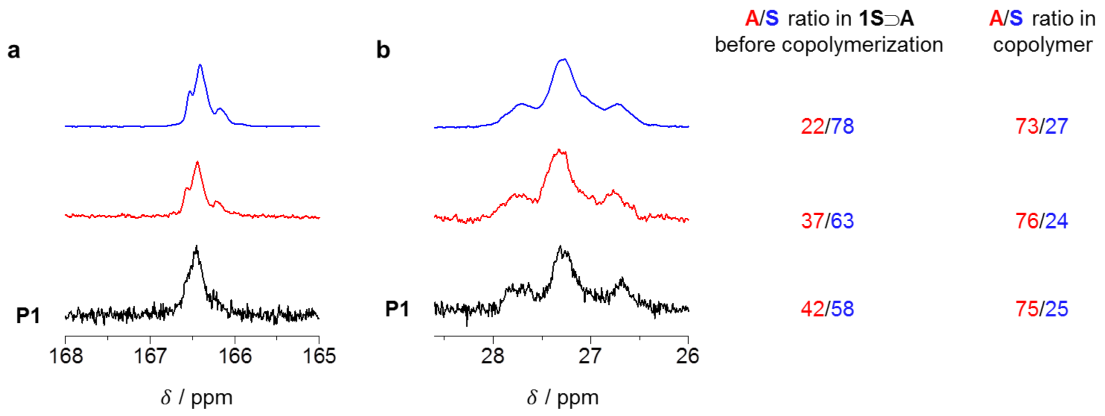

Supplementary Figure 15. ^{13}C NMR spectra of copolymers obtained from **1S**. ^{13}C NMR spectra focusing on (a) carbonyl and (b) methine carbons of copolymers obtained from **1S⊃A** at different initial monomer feed ratios (red and blue lines). The shape and position of these peaks are similar to those of **P1** (Fig. 5), indicating that the copolymers have **ASA**, **AAS** and **AAA** as predominant triads.

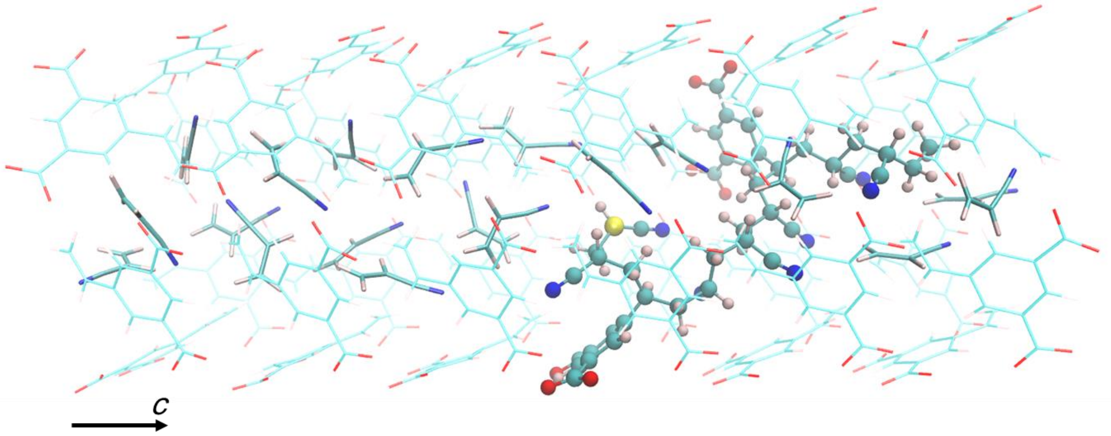

Supplementary Figure 16. Initial structure of the ~**SAA·** model system. **S** and **A** monomers are shown in thin and thick lines, respectively. The Me**AASAAASAA·** chain is shown as a ball and stick model, and the terminal radical is shown as a yellow ball.

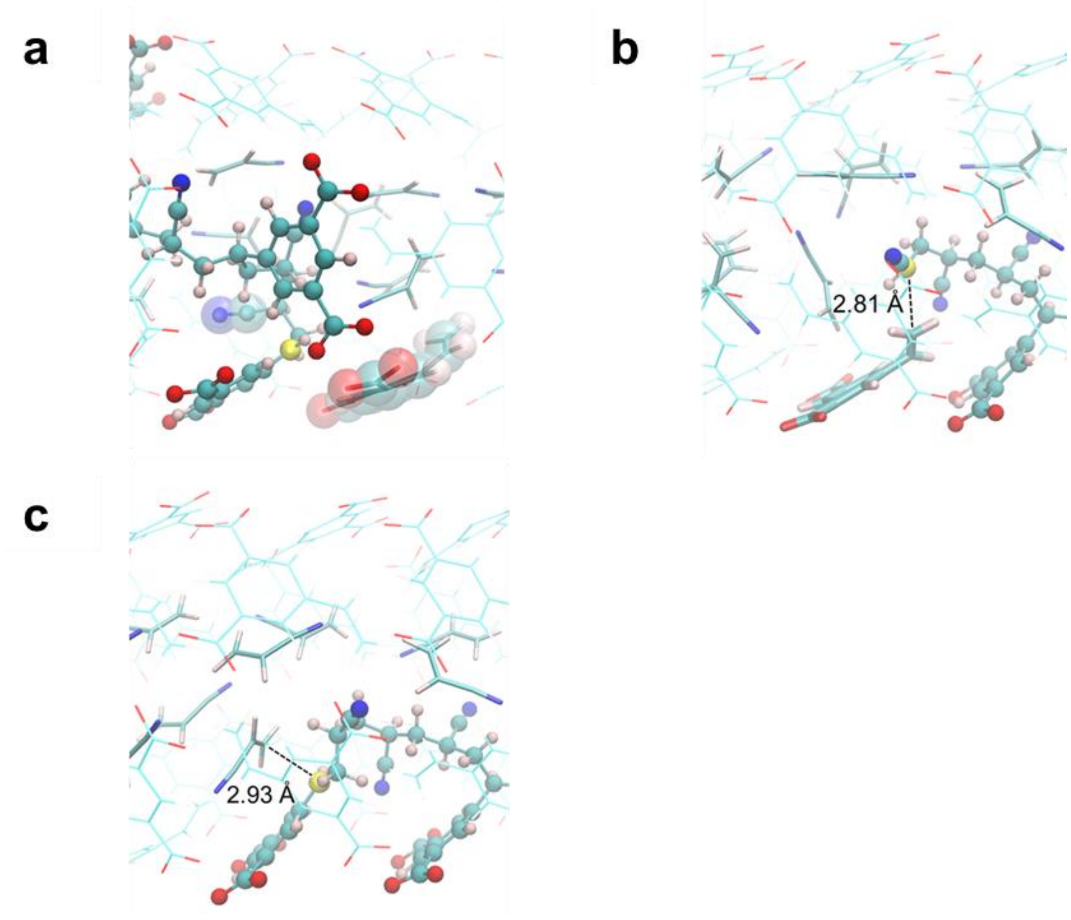

Supplementary Figure 17. Simulation models for **1S⊃A** with propagating radical. (a) Snapshot of the ~**SAAS**· after reacting with the nearest **S** monomer at the adjacent face of the channel. The terminal radical is denoted in yellow. The possibility for this propagation reaction is excluded because the CN group of the penultimate **A** unit and the adjacent **S** moiety (shown as transparent vdW radius spheres) would block contact of the terminal **S** radical with free **A** monomers. Thus, the ~**SAA**· radical is likely to react with **A** to form ~**SAAA**· (Supplementary Fig. 18b). (b) Snapshot of the ~**SAAA**· contacting with the adjacent **S** monomer at the same face of the channel. The distance $r^{R\text{–}M}$ was 2.81 Å, which is the shortest distance that appeared in the REMD simulation at 343 K. This is the preferable formation with less conformational distortion of the chain. Thus, the continuity of the propagation reaction was confirmed only when the radical reacted with the **S** monomer at the same plane of the channel or free **A** monomers. (c) Snapshot of ~**SAAAS**· contacting with a free **A** monomer.

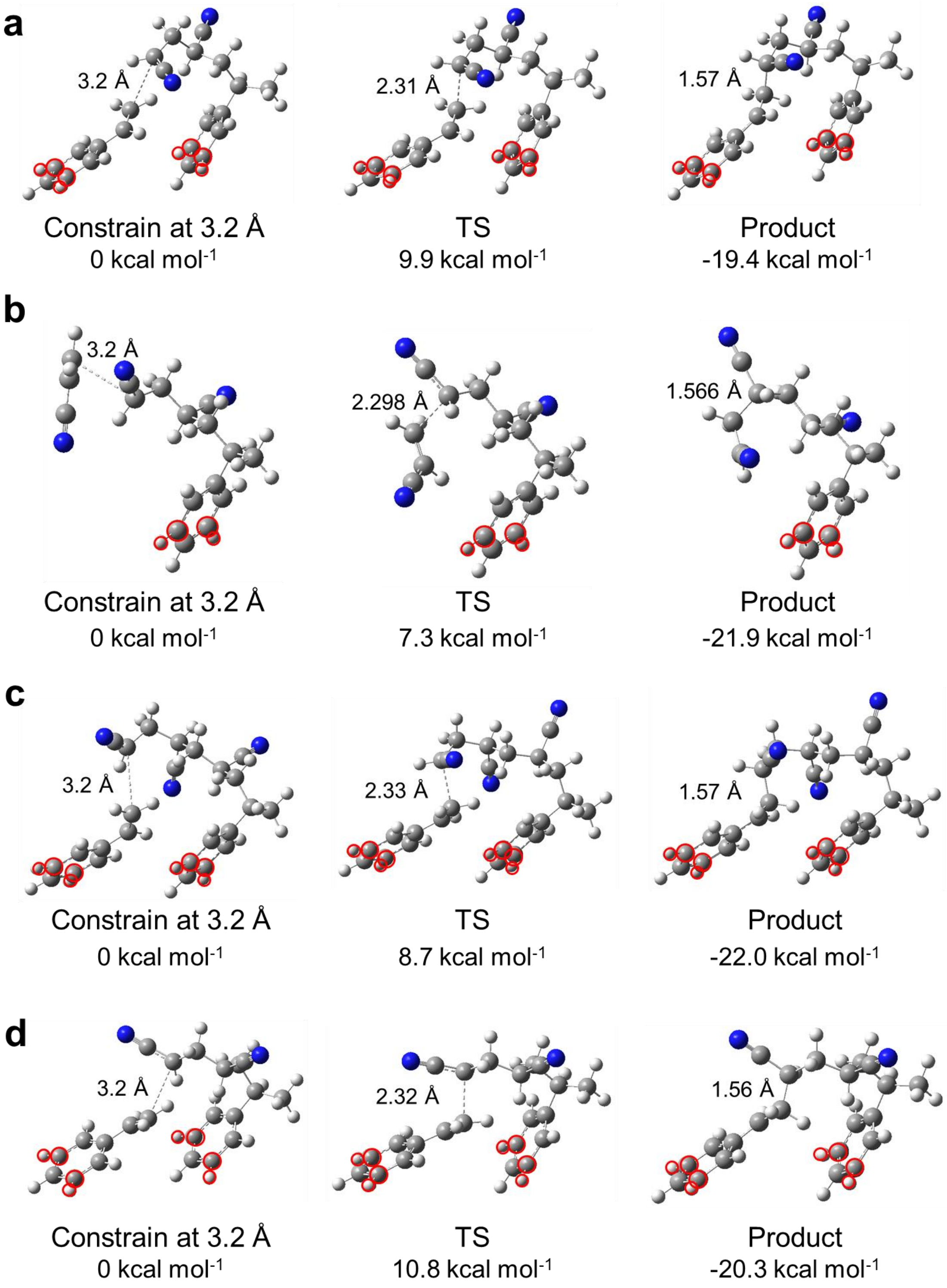

Supplementary Figure 18. Optimized simulation structures during elongation reactions. Optimized structures of (a) ~**SAA**$^{\cdot}$ + **S**, (b) ~**SAA**$^{\cdot}$ + **A** and (c) ~**SAAA**$^{\cdot}$ + **S** elongation reactions by DFT calculations. Atoms with red circles are fixed at their initial position.

The tacticity of the **SA** diad in ~**SAA**$^{\bullet}$ was postulated to be racemo. Reaction barriers (energy difference between the reactant and TS structures) for ~**SAA**$^{\bullet}$ + **S** and ~**SAA**$^{\bullet}$ + **A** were estimated to be 9.9 and 7.3 kcal mol^{-1}, respectively. The barriers for ~**SAAA**$^{\bullet}$ were calculated to be 8.7 and 9.8 kcal mol^{-1} for the addition reactions of **S** and **A** monomers, respectively. In addition, the energies of product structures were always more than 18 kcal mol^{-1} stable compared with those of the reactant. Monomer selectivity of ~**SA**$_n^{\bullet}$ can be estimated from the ratio of kinetic constants,

$$k_X = [C_X \exp(-E_X/RT)],$$

where k_X, C_X and E_X represent the kinetic constant of the addition of radical with monomer X, collision frequency and the reaction barrier, respectively. R represents gas constant (8.314 J K^{-1} mol^{-1}). k_A/k_S reflects the difference in monomer selectivity. In the case of ~**SAA**$^{\bullet}$ (C_A/C_S = 10, E_A = 7.3 kcal mol^{-1}, E_S = 9.9 kcal mol^{-1}, T = 343 K), k_A/k_S was calculated to be 453, indicating that **SAA**$^{\bullet}$ reacts with **A** monomer with very high selectivity (>99%). For ~**SAAA**$^{\bullet}$ (C_A/C_S = 2.0, E_A = 9.8 kcal mol^{-1}, E_S = 8.7 kcal mol^{-1}, T = 343 K), k_A/k_S was found to be 0.40, suggesting that ~**SAAA**$^{\bullet}$ prefers to react with an **S** monomer rather than **A** (68% selectivity). (d) Dependency of monomer selectivity on the tacticity was studied using ~**SAA**$^{\bullet}$ model with meso **SA** diad. In this meso model, REMD simulations revealed that the ratio of **A** to **S** contacting with the radical was 100/1, and reaction barriers for ~**SAA**$^{\bullet}$ + **A** and ~**SAA**$^{\bullet}$ + **S** were calculated to be 8.7 and 10.8 kcal mol^{-1}, respectively, showing the highly preferred formation of **~SAAA**$^{\bullet}$ as well as the racemo diad system.

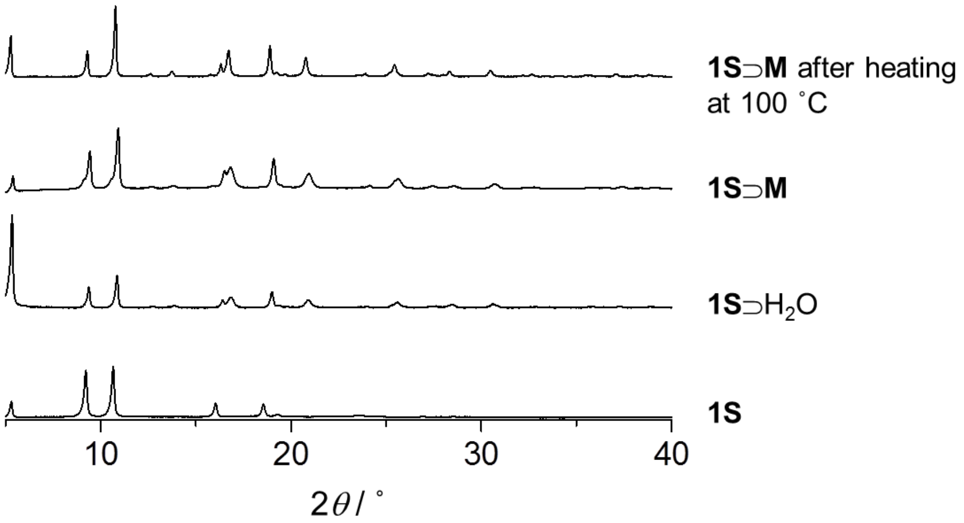

Supplementary Figure 19. XRPD patterns for **1S** with different guests. XRPD patterns for **1S**, **1S**⊃H_2O, **1S**⊃**M**, and **1S**⊃**M** after heating at 100 °C. Similar to the case for the copolymerization using **A** as a guest monomer, the peak positions in the profiles for **1S**⊃**M** before and after the heat treatment are the same as those for **1S**⊃H_2O, showing that the host crystal possessed the same structure as **1S**⊃H_2O during the copolymerization between **M** and **S**.

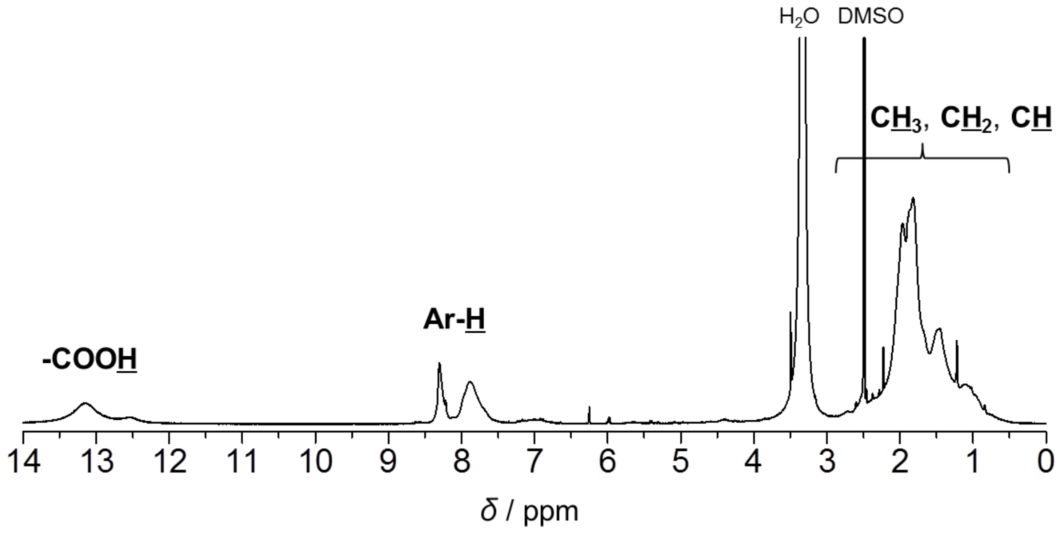

Supplementary Figure 20. ^{1}H NMR spectrum of **P2** in DMSO-d_6. The methyl, methylene and methine groups were assigned using DEPT-90, DEPT-135, HMQC and HSQC NMR spectroscopies (Supplementary Figs. 21–23). The copolymer composition of **P2** could be determined by comparing the relative integrations of the aromatic protons with aliphatic protons (**M**/**S** = 76/24).

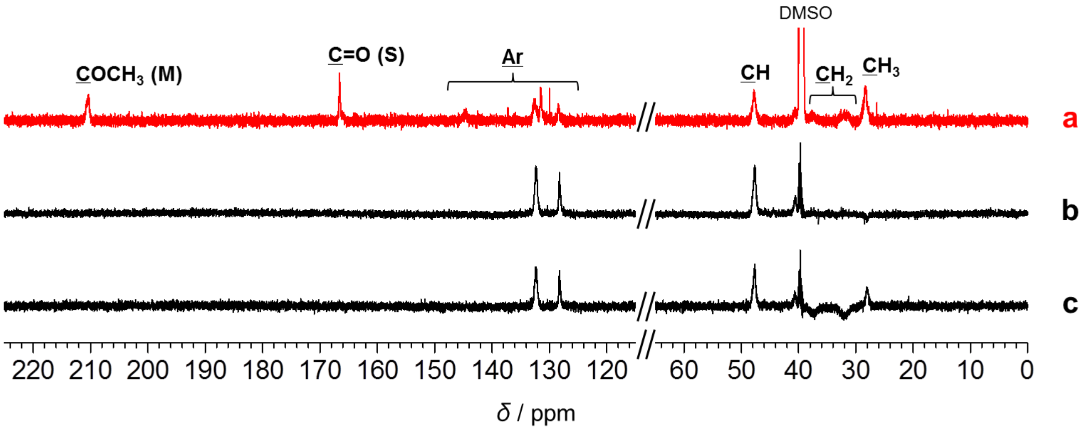

Supplementary Figure 21. (a) ^{13}C NMR, (b) DEPT-90 and (c) DEPT-135 spectra of **P2**. In DEPT-135, negative peaks are assignable to CH_2, while positive peaks correspond to CH or CH_3. Only CH signals are observed as positive peaks in DEPT-90. The aromatic and carbonyl carbons were assigned by the chemical shifts.

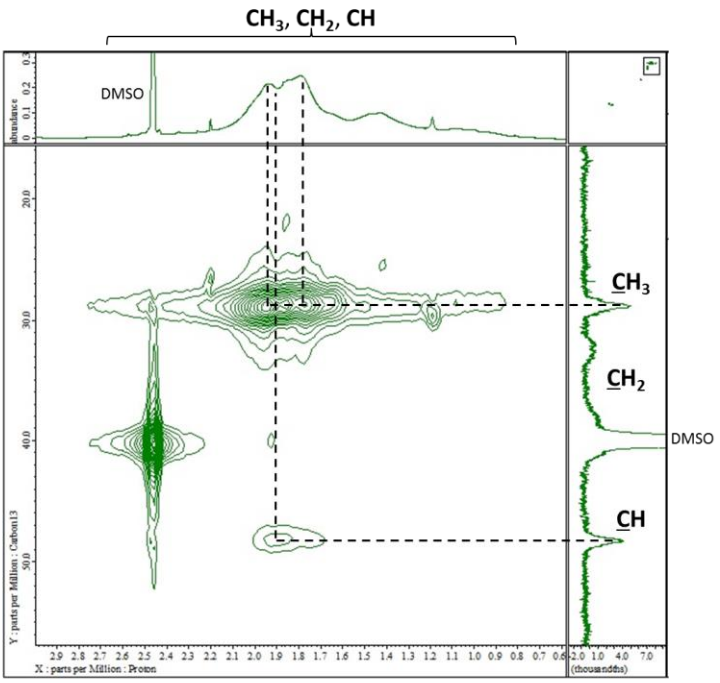

Supplementary Figure 22. HMQC spectrum of **P2**. Cross peaks for methyl and methine protons were observed.

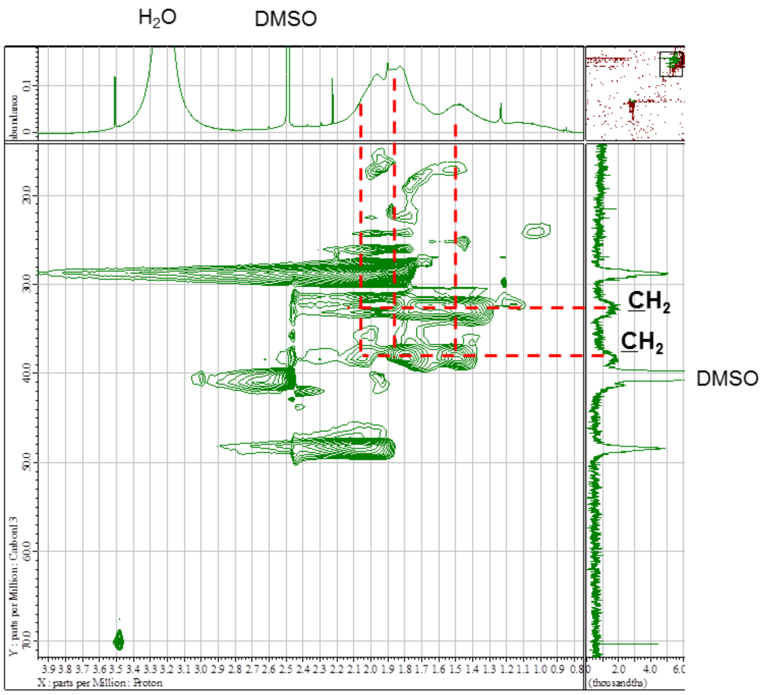

Supplementary Figure 23. HSQC spectrum of **P2**. In contrast to the HMQC spectrum, cross peaks for methylene protons were observed.

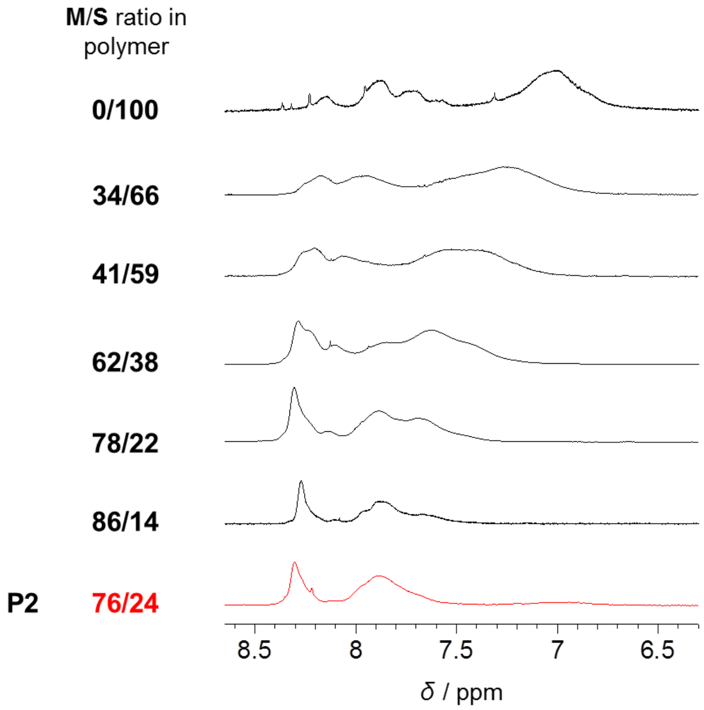

Supplementary Figure 24. Comparison of the ^{1}H NMR spectra of **P2** and random copolymers. As is the case with **A**-**S** copolymer, the peaks for aromatic protons were shifted towards lower magnetic field with decreasing **S** units in the **M**-**S** copolymer. **P2** did not show a peak at 7.65 ppm, which was observed in random copolymers with similar composition, suggesting a less bulky environment around the **S** unit in **P2**.

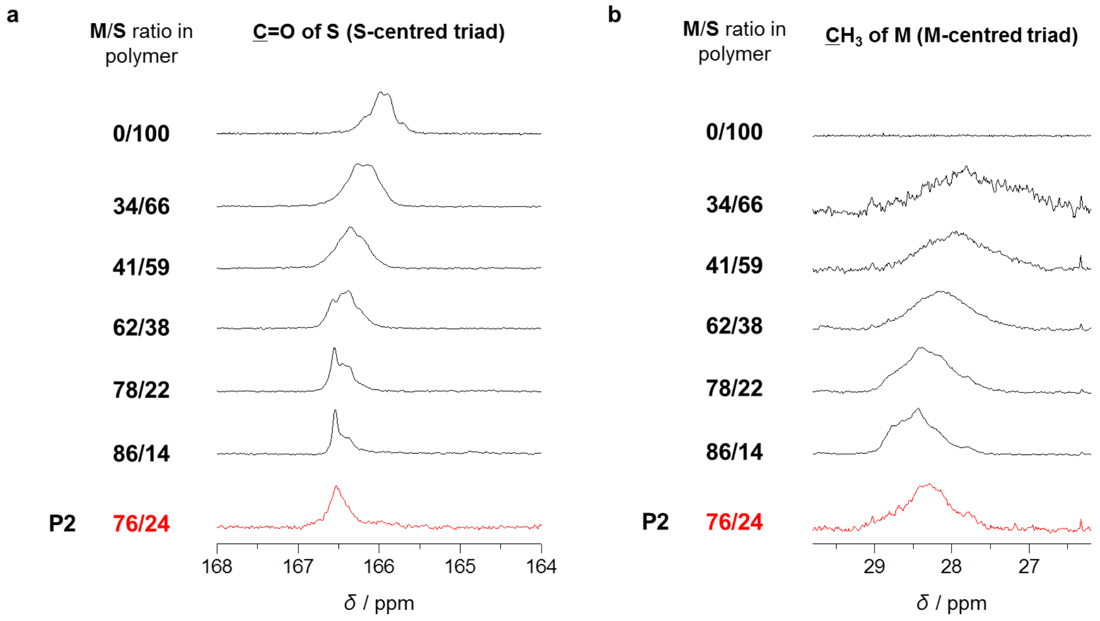

Supplementary Figure 25. Structural characterization of polymers using ^{13}C NMR. ^{13}C NMR spectra of **P2** and polymers synthesized in DMF, focusing on (a) carboxyl carbon of **S** and (b) methyl carbon of **M** in the polymers, from which **S**- and **A**-centred triads can be analysed, respectively. For **S**-centred triad: in the spectrum of **P2** (**M**/**S** = 76/24), only a single peak was observed at 166.5 ppm, while there were two major signals in the spectra of random copolymers with similar **M/S** compositions. This implies homogeneity of the monomer sequence and the highly isolated environment of the **S** unit among **M** unit chains in **P2**, forming **MSM** triads. For the **M**-centred triad: the peak top was shifted to lower field with decreasing **S** ratio in the copolymer. Observation of almost identical peak shapes in the random copolymer (**M**/**S** = 78/22) and **P2** (**M**/**S** = 76/24) indicates that **P2** would have **MMM** and **MMS** as predominant triad sequences.

Supplementary References

1. Xu, Y. *et al.* Effects of mesogenic shape and flexibility on the phase structures of mesogen-jacketed liquid crystalline polymers with bent side groups containing 1,3, 4-oxadiazole. *Macromolecules* **42,** 2542–2550 (2009).
2. Spek, A. L. *PLATON 99, A Multipurpose Crystallographic Tool* (Utrecht University, Utrecht, The Netherlands, 1999).
3. Spek, A. L. PLATON SQUEEZE: a tool for the calculation of the disordered solvent contribution to the calculated structure factors. *Acta Cryst. C* **71,** 9–18 (2015).
4. Sato. H, *et al.* Self-accelerating CO sorption in a soft nanoporous crystal. *Science* **343,** 167–170 (2014).
